# Supplementary material for: Predatory Dogs as Drivers of Social Behavior Changes in the Central Himalayan Langur (Semnopithecus schistaceus) in Agro-Forest Landscapes
Source: Biology (Basel). 2024 Jun 4;13(6):410. doi: 10.3390/biology13060410 (PMC11200765; doi:10.3390/biology13060410)
Supplement: Supplementary file 1 [file biology-13-00410-s001.zip › biology-3001892-supplementary.pdf]

Supplementary Table S1. Classification of open forests within the S Group territory (See Figure 1).

**Location Name**	**Description**
**Gweer Alnus Patch**	Situated along the river in the lower portion of Gweer Village, this location has a small number of *Alnus nepalensis* trees, which provide the only canopy in the region. This area is surrounded by the human settlements and agricultural land.
**Gweer Prunus Patch**	The eastern section of the S Group territory is situated within Gweer Village. Characterized by sparse tree coverage, with only 4-6 *Prunus cerasoides* trees providing connectivity, this area is surrounded by the human settlements and agricultural land.
**Kunkuli School**	Situated at the southwest corner of the S Group territory, this open area is predominantly occupied by human settlements. Despite the sparse presence of trees, including *Prunus cerasoides, Celtis australis*, and *Grewia optiva* around the village houses, it remains highly preferred by the langurs.
**Kunkuli Agriculture Land**	This area encompasses agricultural fields in Kunkuli village and dominated primarily by *Celtis australis, Ficus virens,* and *Grewia optiva* trees planted along the edges of the agricultural land.
**Mandal Shivalay**	The western part of the S Group territory has open agricultural fields on one side and a river on the other. Alongside the riverbank, a row of *Prunus cerasoides* trees stands prominently, serving as the primary attraction for the langurs.
**Siroli Bridge**	This area includes patches of *Quercus leucotrichophora*, encircling the village of Siroli. It is also characterized by human settlements and agricultural fields.
**Siroli Agricultural Land**	This area encompasses agricultural fields in Siroli village and dominated primarily by *Celtis australis* and *Grewia optiva* trees planted along the edges of the agricultural land.

Supplementary Table S2. Classification of dense forests within the S Group Territory (See figure 1).

**Location Name**	**Description**
**Forest Chowki**	The dense *Quercus leucotrichophora* forest area which is the central point of the S Group territory. While one side extends into continuous forest, the other three sides are bordered by human settlements and agricultural land.
**Gondi Cliff**	This area is an extrema northwestern part of the S Group territory situated in the Gondi village area. This landscape is dominated by the *Quercus leucotrichophora* forest.
**Kunkuli Kaula Patch**	Located to the north of Kunkuli village and running parallel to the national highway, it's a dense forested area dominated by *Daphniphyllum himalense*.
**Kunkuli Oak Patch**	The largest contiguous dense forest dominated by *Quercus leucotrichophora* within the S Group territory stretches from the southern edge of their territory all the way to Kunkuli Village.
**Kunkuli Shivalay**	It comprises dense *Quercus leucotrichophora* forests, situated in the southeastern region of the S Group territory. These forests are connected to the Shiva temple in Kunkuli village and stretch all the way to Makroli village.
**Siroli Road Oak Patch**	This is the northern part of the S Group territory lies in the Siroli Village. This area is dominated by the dense *Quercus leucotrichophora* continues forest.
**Siroli Cliff**	This area lies at the northernmost edge of the S Group territory and dominated by dense *Quercus leucotrichophora*.
